# Cortical spreading depression and meningeal nociception

**DOI:** 10.1016/j.ynpai.2022.100091

**Published:** 2022-04-22

**Authors:** Simone Carneiro-Nascimento, Dan Levy

**Affiliations:** Department of Anesthesia, Critical Care and Pain Medicine, Beth Israel Deaconess Medical Center and Harvard Medical School, Boston, MA 02115, United States

**Keywords:** Migraine, Cortical spreading depression, Meningeal nociception, ATP, adenosine triphosphate, Ca^2+^, calcium ions, CCL2, C–C motif chemokine ligand 2, COX, cyclooxygenase, CSD, cortical spreading depression, CSF, cerebrospinal fluid, HMGB1, high mobility group box protein 1, IL-1β, interleukin 1 beta, IL-6, interleukin 6, K^+^, potassium ions, K(ATP), ATP-sensitive potassium channels, KCl, potassium chloride, NaCl, sodium chloride, NK-1, neurokinin-1, NMDA, N-methyl-D-aspartate, TNC, trigeminal nucleus caudalis, TG, trigeminal ganglion

## Abstract

Migraine results in an enormous burden on individuals and societies due to its high prevalence, significant disability, and considerable economic costs. Current treatment options for migraine remain inadequate, and the development of novel therapies is severely hindered by the incomplete understanding of the mechanisms responsible for the pain. The sensory innervation of the cranial meninges is now considered a key player in migraine headache genesis. Recent studies have significantly advanced our understanding of some of the processes that drive meningeal nociceptive neurons, which may be targeted therapeutically to abort or prevent migraine pain. In this review we will summarize our current understanding of the mechanisms that contribute to the genesis of the headache in one migraine subtype – migraine with aura. We will focus on animal studies that address the notion that cortical spreading depression is a critical process that drives meningeal nociception in migraine with aura, and discuss recent insights into some of the proposed underlying mechanisms.

## Highlights

• CSD evoked persistent activation and mechanical sensitization of dural nociceptors is likely to drive the headache phase in migraine with aura.
• The development of neurogenic-mediated dural vasodilatation and increased plasma protein extravasation in the wake of CSD may not contribute to meningeal nociception.
• Cortical vasoconstriction and reduced oxygen availability following CSD do not contribute to meningeal nociception.
• Cortical neuroinflammation, involving neuronal pannexin1 and calcium-independent astrocytic signaling drive meningeal nociception following CSD.
• CSD-related closing of K(ATP) channels and release of COX-driven prostanoids mediate the activation and sensitization of dural nociceptors respectively.

Migraine is considered one of the most prevalent neurological disorders, affecting about 15% of the adult population worldwide (Global Burden of Disease Study, 2017) and the leading cause of disability in under 50s (Steiner et al., 2018). The biological origin of migraine remains unclear. However, it is now well accepted that the head pain during a migraine attack is mediated by increased activity in primary afferent nociceptive neurons that innervate the cranial meninges (Ashina et al., 2019, Levy, 2012, Olesen et al., 2009). Although the endogenous processes that drive meningeal nociception during a migraine attack remain unclear, a key theory suggests a local inflammatory process triggered by exogenous or endogenous factors (Ashina et al., 2019, Levy et al., 2019). One consistent finding across all migraine patients is the presence of cortical hyperexcitability (Brennan and Pietrobon, 2018, de Tommaso et al., 2014, Maniyar et al., 2014a, Maniyar et al., 2014b, Tolner et al., 2019, Vecchia and Pietrobon, 2012, Zielman et al., 2017). In a subset of patients and attacks, such cortical hyperexcitability is believed to drive episodes of cortical spreading depression (CSD) – a massive concentric wave of neuronal and astroglia depolarization, followed by a transient depression of cortical synaptic activity, that mediates the aura phase (Pietrobon and Moskowitz, 2013). The temporal proximity between the aura phase and the onset of the headache in migraine has led several investigators, almost four decades ago, to propose a link between the aura and the headache phases (Blau, 1984, Moskowitz, 1984, Lauritzen, 1985), and further prompted basic research to examine a link between CSD and migraine pain. Here, we will summarize preclinical data that support the notion that CSD is a critical process that drives meningeal nociception in migraine with aura, and discuss recent insights into some of the proposed underlying mechanisms.

## Indirect evidence linking CSD and meningeal nociception

Moskowitz and colleagues (Moskowitz et al., 1993) were the first to address the idea that CSD is a major endogenous event that drives the meningeal sensory system in a rat model. Using c-fos as a molecular marker of neural activation, they demonstrated an association between a series of CSD episodes evoked by cortical microinjections of hyperosmolar potassium chloride (KCl) and the activation of neurons in the trigeminal dorsal horn, which process nociceptive afferent input from the head. The additional observation that the increase in trigeminal c-fos expression was inhibited by transecting the nasociliary nerve, a major sensory nerve that supplies the intracranial dura mater, led this group to suggest that CSD is a noxious event that can activate the migraine pain pathway. Ingvardsen et al. (1997) argued against a causative role for CSD in driving the trigeminal pain in migraine. Using a similar approach, they reported a correlation between the number of KCl stimuli used to evoke the CSD events and the trigeminal c-fos expression, but not with the number of CSDs produced, suggesting that the stimulus in itself rather than the CSD events is responsible for the trigeminal activation. The concentration of KCl used in these studies can indeed activate dural nociceptors (Strassman et al., 1996). However, Moskowitz et al. also included a control arm in their study that tested the effect of sodium chloride (NaCl) stimulation, which can also trigger dural nociceptors (Strassman et al., 1996), and found it to be less efficient in producing TNC c-fos (Moskowitz et al., 1993), thus supporting their proposed link between CSD and the trigeminal ganglion (TG) activation observed.

Bolay and colleagues (Bolay et al., 2002) provided, almost a decade later, the first indirect evidence that CSD can trigger activity in dural nociceptors. In that study, a single CSD, evoked using a pinprick stimulus in a rat’s cortex, produced a prolonged increase in dural blood flow associated with increased plasma protein leakage, both of which were abrogated by surgical deafferentation of the intracranial dura. CSD-evoked prolonged dural vasodilation was later replicated in a mouse model (Karatas et al., 2013). Of note, similar to the provocation of CSD using local application of hyperosmolar KCl, a pinprick (mechanical) stimulus can also activate dural nociceptors and potentially enhance their response properties by causing dural injury. However, the contribution of these factors to the emergence of persistent activation of dural nociceptors in that study was likely minimal given that CSD-related meningeal vascular response was inhibited in animals pretreated with the N-methyl-D-aspartate (NMDA) receptor antagonist MK801, which blocked the CSD propagation (Bolay et al., 2002).

## Direct evidence that CSD can drive the meningeal sensory system

Using single-unit recording from TG neurons in a rat model, we demonstrated that CSD could produce dural nociceptor activation (Zhang et al., 2010). We found that a single CSD event leads to a two-fold increase in the activity of about half of the nociceptors tested, an effect that lasted for approximately one hour. In that study, CSD was associated with several patterns of dural nociceptor responses: acute nociceptor discharge restricted to the brief CSD event; a prolonged activation that starts during the CSD event; and a biphasic pattern of activation, involving acute discharge, followed by a delayed and prolonged activation that commences 10–20 min after the CSD event. The possibility that stimuli used to induce CSD, which were administered near the nociceptors’ receptive field, contributed to the post-CSD nociceptive responses was not directly addressed in that study. However, the finding that similar patterns of dural nociceptor activation emerged regardless of the method used to elicit the CSD event (i.e., pinprick, topical KCl application, or electrical stimulation of the cortex), suggest that CSD itself, rather than the method used to produce it, drives dural nociceptors. In a later study, we were able to exclude the possibility that the method used to elicit CSD in itself influences the activity of dural nociceptors: we showed comparable delayed and prolonged dural nociceptor activation patterns when CSD was triggered remotely and then propagated under the receptive field of the recorded nociceptors (Zhao and Levy, 2015). Using this modified preparation, we further found that the propensity to develop prolonged nociceptor activation following CSD is related to the presence of basal ongoing activity, but neither to the transient activation during the CSD phase nor to the nociceptors’ responsiveness to inflammatory mediators. We also found an inverse correlation between the onset of the prolonged nociceptor activation and the number of their dural receptive fields. Thus, from a mechanistic approach, the prolonged meningeal nociceptive responses following CSD may not be related to the chemosensitivity of the nociceptors but rather to other properties such as basal ongoing activity and the number of dural terminal arborizations. The presence of basal ongoing activity before CSD induction may be a possible indicator of nociceptor sensitization that develops in response to the surgical exposure in these studies, which causes a mild meningeal inflammatory response (Levy et al., 2007). Whether such surgery-related nociceptor sensitization is a prerequisite to developing a prolonged nociceptor response following CSD, similar to a nociceptive priming effect (Burgos-Vega et al., 2016), remains unclear. The shorter delay in activation after CSD in nociceptors with more receptive fields may be related to a spatial summation mechanism that facilitates the onset of the persistent nociceptors' activation.

In addition to increased ongoing activity, increased mechanosensitivity (i.e., sensitization) of dural nociceptors is also considered to play a key role in driving migraine pain, in particular, the exacerbation of the headache during conditions that momentarily increase intracranial pressure, such as head movements and coughing (Strassman and Levy, 2006, Strassman et al., 1996). Using a rat model, we discovered that CSD also provokes a pronounced and persistent mechanical sensitization of dural nociceptors (Zhao and Levy, 2016). Importantly, that study showed that the sensitization and increased ongoing activity following CSD are not correlated. We also found that the sensitizing effect of CSD generally lasts longer than the increase in ongoing activity, suggesting that CSD-evoked mechanical sensitization of dural nociceptors may play a more substantial role in the development of the headache phase during a migraine attack.

To examine whether the CSD-evoked dural nociceptors responses are sufficient to drive the central migraine pain pathway, we also recorded in a rat model CSD-evoked responses of second-order neurons located in the medullary dorsal horn that receive convergent input from the dura and cephalic skin (Zhang et al., 2011). In that study, a single CSD was associated with neural activation patterns similar to those observed in dural nociceptors, including immediate and delayed activation. Melo-Carrillo and colleagues (Melo-Carrillo et al., 2017a) further demonstrated that CSD leads to sensitization of central trigeminal nociceptive neurons. However, this effect was limited to high-threshold dorsal horn neurons, which do not respond to innocuous mechanical stimulation of the skin at baseline (Melo-Carrillo et al., 2017a). That study also found that systemic administration of an anti-calcitonin gene-related peptide (CGRP) antibody, used in migraine prophylaxis, inhibited the development of both the activation and sensitization of high threshold trigeminal neurons following CSD, further substantiating the link between CSD, meningeal nociception, and migraine headache.

## CSD, meningeal nociception, and migraine-like pain behaviors

A long-standing problem with the notion that CSD drives meningeal nociception has been the conflicting preclinical evidence that CSD leads to pain behaviors (Bogdanov et al., 2013, Filiz et al., 2019, Fioravanti et al., 2011, Harriott et al., 2021, Houben et al., 2017, Karatas et al., 2013). One critical confounder in many of these studies was using hyperosmolar KCl as the local triggering stimulus (Bogdanov et al., 2013, Filiz et al., 2019, Fioravanti et al., 2011, Karatas et al., 2013). Furthermore, prolonged exposure to KCl gives rise to multiple CSD events, which may not be clinically relevant to migraine. While KCl-evoked CSD has been shown to produce mechanical allodynia, a common sensory finding in migraine, the induction of CSD using a pinprick stimulation failed to do so (Fioravanti et al., 2011), arguing against the ability of a single CSD to evoke sufficient meningeal nociception that drives migraine pain. A recent study by Harriott et al. (2021) overcame these limitations by eliciting CSD non-invasively using a transcranial optogenetic approach in a mouse model. In that study, a single CSD produced in one side of the cortex produced bilateral cephalic allodynia. While ipsilateral head pain is mainly observed in migraine attacks with aura, allodynia can initially develop ipsilateral to the side of head pain and later spreads to the contralateral side (Burstein et al., 2000). Optogenetic induction of a single CSD, however, failed to produce a grimacing behavior indicative of pain, contrary to what has been observed in response to multiple CSD events produced by repeated KCl stimulation (Karatas et al., 2013). Finally, while a single CSD event induced using the optogenetic approach failed to elicit an anxiety behavior, multiple CSD events triggered optogenetically or using a KCl stimulus (Bogdanov et al., 2013) produced it. Our finding that repeated CSD events give rise to similar patterns of dural nociceptor activation as observed after a single event (Zhao and Levy, 2015) suggests that the exaggerated behavioral response following multiple CSD may occur independently of the meningeal nociceptive effect of CSD, potentially by affecting cortical and subcortical circuits.

## Is there a role for meningeal neurogenic inflammation in mediating meningeal nociception following CSD?

**Fig. 1:**
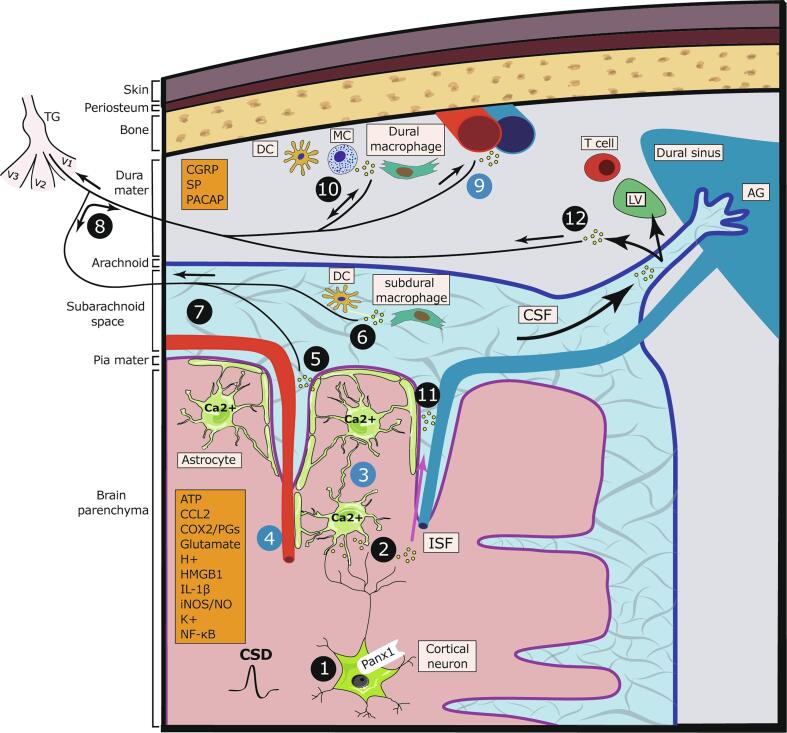
Cortical to meninges signaling and CSD-evoked meningeal nociception. CSD is associated with cortical and meningeal events that lead to the release of several mediators (orange boxes); these events and related mediators ​may (black circles) or may not (blue circles) drive meningeal nociception. (1) Cortical neuronal activation leads to pannexin-1 channel opening and caspase-1 activation, followed by parenchymal HMGB1 and IL-1β release. Cortical neuronal activation also leads to the release of ATP, K^+^, H^+^, and glutamate (2) Neuron-to-astrocyte signaling leading to NF-kB activation and COX-2 and iNOS upregulation with prostaglandins and NO release. (3) Astrocyte calcium wave and release of prostanoids (4) drive cortical vasoconstriction and reduction in tissue oxygen tension. (5) Activation ​of glia limitans ​with the release of pronociceptive mediators into the subarachnoid space that leads to (6) activation of subdural meningeal immune cells and (7) Leptomeningeal afferent nerve endings. (8) Antidromic axon reflex leads to the release of sensory neuropeptides from collateral dural nerve endings, which directly or indirectly produces (9) dural vasodilation and increased capillary permeability. (10) Nociceptor-evoked activation of dural immune cells, leading to the production of inflammatory mediators and reciprocal activation and sensitization of dural nociceptors. (11) Delayed clearance of parenchymal inflammatory mediators into the CSF-filled subarachnoid space due to closure of perivascular space and reduced glymphatic flow. (12) Delayed activation of dural nociceptors with nerve endings near the dural sinuses by CSF mediators that egress from arachnoid granulations before entering dural lymphatic ​vessels.​ Abbreviations: ATP, adenosine triphosphate; AG, arachnoid granulations; CCL2, C–C motif chemokine ligand 2; CGRP, calcitonin gene-related peptide; COX-2, cyclooxygenase-2; CSD, cortical spreading depression; CSF, cerebrospinal fluid; DC, dendritic cell; HMGB1, high mobility group box protein 1; IL-1β, interleukin 1 beta; iNOS, inducible nitric oxide synthase; ISF, interstitial fluid; LV, lymphatic vessel; MC, mast cell; NF-kB, nuclear factor kappa B; NO, nitric oxide; PACAP, pituitary adenylate cyclase-activating polypeptide; Panx1, pannexin-1; PGs, prostaglandins; SP, substance P; TG, trigeminal ganglion; V1, ophthalmic nerve; V2, maxillary nerve; V3, mandibular nerve.

CSD is associated with the parenchymal release of numerous molecules with nociceptive properties, including adenosine triphosphate (ATP) (Schock et al., 2007), glutamate (Zhou et al., 2013), potassium ions (K^+^) (Enger et al., 2015), and protons (Csiba et al., 1985). A key migraine theory (Bolay et al., 2002, Karatas et al., 2013) proposes that these mediators diffuse outward towards the leptomeninges overlaying the affected cortical region, resulting in the activation of nociceptive afferent nerve endings localized to leptomeninges (Fontaine et al., 2018; Fricke et al., 1997)*.* This leads to an axon reflex and antidromic release of proinflammatory neuropeptides (e.g., CGRP, substance P) from collateral dural nerve endings and the subsequent development of neurogenic inflammation, including dural vasodilation and increased vascular permeability (Fig. 1). Subsequently, this sterile inflammatory response promotes the release of additional nociceptive molecules that cause the prolonged wave of dural nociceptor excitation and sensitization. Supporting this theory is the anatomical finding of trigeminal sensory neurons with branching axons that innervate the dural and leptomeningeal vascular structures (i.e., axonal projections to the middle meningeal artery and the middle cerebral artery) (O'Connor and van der Kooy, 1986). These axonal branching points were suggested to occur at a proximal site just after leaving the trigeminal cell body giving rise to different innervation routes (Fig. 1). However, this pattern of primary afferent branching contrasts with the distal branching at the tissue level that is thought to mediate neurogenic inflammation in neighboring, unstimulated cutaneous tissue (Chiu et al., 2012). At present, very little is known about the response properties of leptomeningeal afferents, so it is unclear whether they can respond to the cortical mediators released during the CSD and drive dural neurogenic inflammation. The findings that treatment with triptans and CGRP receptor antagonists, which abort migraine headaches, can inhibit meningeal neurogenic inflammation provide further support for the neurogenic inflammatory theory of migraine. However, these anti-migraine agents can abort migraine pain through different mechanisms, for example, by acting at the trigeminal dorsal horn levels to disrupt the communication between central terminals of dural nociceptors and the trigeminal second-order dorsal horn neurons (Levy et al., 2004, Storer et al., 2004).

Despite being considered a mechanism of meningeal nociception and migraine pain, there is very little evidence that neurogenic inflammation can drive nociception in other tissues (Reeh et al., 1986). We recently addressed the possibility that CSD-evoked meningeal nociception involves an axon reflex. In that study, we have shown that a brief stimulation of dural nociceptors with KCl gives rise to a CGRP-dependent prolonged activation of the same afferents (Zhao and Levy, 2018a). However, this response was not accompanied by mechanical sensitization, suggesting that the development of neurogenic inflammation following CSD, if it occurs, may not drive the entire repertoire of the meningeal nociceptor responses. Peripheral CGRP release is a key mediator of meningeal neurogenic inflammation, but whether its local action is sufficient to generate meningeal nociception, including in response to CSD, is controversial. We have shown in male rats that dural application of CGRP, which drives potent dural vasodilatation, is not sufficient to activate or sensitize dural nociceptors (Levy et al., 2005). We also found that blockade of meningeal CGRP receptors using olcegepant (BIBN4096) does not inhibit the CSD-evoked prolonged activation and mechano-sensitization of meningeal dural nociceptors in male rats (Zhao and Levy, 2018a). However, a recent behavioral study in rats and mice has shown that local CGRP action can exert a meningeal nociceptive response but only in females (Avona et al., 2019). Whether CGRP is released in the meninges following CSD and drives dural neurogenic vasodilation also remains questionable (Piper et al., 1993, Ebersberger et al., 2001, Schain et al., 2019). The finding that peripheral sequestering of CGRP with a monoclonal antibody can reduce the propensity of A-delta dural nociceptors (but not C-nociceptors) to become activated following CSD (Melo-Carrillo et al., 2017b) raises the possibility that basal CGRP level somehow modulates the responsiveness of this dural afferent subpopulation to CSD. Recent work demonstrated CGRP-receptor expression by several meningeal immune cells, including macrophages, dendritic cells, mast cells, B cells, and T cells (Van Hove et al., 2019), suggesting that meningeal CGRP elaboration following CSD, if it occurs, may somehow contribute to meningeal nociception by modulating immune cell function, rather than via its vascular action (Fig. 1). Whether CGRP produces a proinflammatory immune response, nonetheless, is context-dependent. For example, CGRP release following nociceptors stimulation promotes the recruitment and activation of dendritic cells and T cells and the production of proinflammatory cytokines in cutaneous inflammation (Cohen et al., 2019). On the other hand, nociceptors’ release of CGRP suppresses the recruitment of neutrophils and T cells and the related production of proinflammatory cytokines during host immune response (Baral et al., 2018, Chiu et al., 2013, Pinho-Ribeiro et al., 2018).

Could other mediators released from peripheral terminals of activated dural nociceptors drive neurogenic inflammation and potentially dural nociception following CSD? Release of substance P from dural nociceptors and activation of its canonical receptor neurokinin-1 (NK-1) have been shown to drive the CSD-evoked dural vasodilation and plasma protein extravasation (Bolay et al., 2002). However, there is little support for NK-1 related signaling in migraine pain (Diener, 2003, Goldstein et al., 1997), questioning the role of substance P-induced meningeal neurogenic inflammation as a mechanism underlying migraine pain. Substance P, however, can promote inflammation and nociceptive responses independent of the NK-1 receptor via the activation of Mas-related G protein-coupled receptors expressed on mast cells (Green et al., 2019) and the nociceptors themselves (Azimi et al., 2017, Azimi et al., 2016). Activity-dependent release of mediators from nonpeptidergic dural nociceptors may also play a role. Of potential interest is high mobility group box protein 1 (HMGB1), which can be released from activated nociceptive afferents and promote inflammation (Yang et al., 2021). Activation of nonpeptidergic nociceptors and the subsequent release of glutamate from their peripheral nerve ending, however, could also interfere with neurogenic inflammation by suppressing the activation of mast cells (Zhang et al., 2021).

## Cortical vascular and metabolic changes may not drive meningeal nociception in the wake of CSD

Migraine with aura is associated with cortical hemodynamic changes, including a sustained reduction in cortical blood flow contemporaneous with the headache phase (Hadjikhani et al., 2001, Lauritzen and Olesen, 1984, Olesen et al., 1981). In rodent models, CSD also leads to prolonged cortical hypoperfusion that can last up to 2 h (Ayata and Lauritzen, 2015). Studies in rodents also demonstrated transient cortical hypoxia followed by a prolonged and milder reduction in cortical tissue partial pressure of oxygen (tpO_2_), and hemoglobin desaturation, concomitant with the cortical hypoperfusion phase (Chang et al., 2010, Piilgaard and Lauritzen, 2009, Takano et al., 2007). The mechanisms responsible for these metabolic responses are likely numerous, although the release of cyclooxygenase (COX)-derived vasoconstricting prostanoids and other eicosanoid metabolites of arachidonic acid plays a key role (Fordsmann et al., 2013, Gariepy et al., 2017, Shibata et al., 1992). Reduced blood flow and tissue oxygenation can trigger a migraine attack in susceptible individuals (Appenzeller, 1994, Arngrim et al., 2016, Broessner et al., 2016, Frank et al., 2020, Schoonman et al., 2006). These findings, together with the notion that reduced blood flow and tissue oxygenation lead to the sensitization of nociceptors in other tissues (Hillery et al., 2011, MacIver and Tanelian, 1992, Mense and Stahnke, 1983), raises the possibility that the mechanisms responsible for driving dural nociceptors in the wake of CSD involve similar vascular and metabolic changes. We recently investigated this notion by recording the activity of dural nociceptors along with changes in cortical blood flow and tissue oxygenation following CSD (Zhao and Levy, 2018b). We have shown that cortical hypoperfusion and decreased oxygen availability coincide with the emergence of the prolonged activation and sensitization of dural nociceptors. However, while the COX-inhibitor naproxen ameliorated these CSD-evoked metabolic changes, it did not affect the activation of the dural nociceptors. Thus, there is likely a dissociation between these CSD-related cortical metabolic perturbations, or downstream processes, and the mechanism responsible for driving activity in dural nociceptors. It further questions the contribution of locally released prostanoids and related inflammatory responses in mediating the CSD-evoked prolonged activation of dural nociceptors. The findings that COX inhibition attenuates the CSD-evoked dural vasodilation and activation of pial and dural macrophages (Schain et al., 2020) additionally question the link between the vascular components of neurogenic inflammation, activation of meningeal immune cells, and dural nociceptor activation in the wake of CSD. That naproxen blocks the activation of dural nociceptors following meningeal stimulation with a mixture of mediators found in inflammatory exudates (i.e., inflammatory soup) (Levy et al., 2008) points to the possibility that CSD drives activity in dural nociceptors via a different inflammatory mechanism.

While COX-derived prostanoids may not promote the CSD-evoked prolonged activation of dural nociceptors, we recently demonstrated COX involvement in mediating their mechanical sensitization (Zhao and Levy, 2018b). The mechanism by which COX-derived mediators promote the sensitization of dural nociceptors following CSD remains unclear. It is, however, unlikely to involve cortical hypoperfusion and decreased oxygen availability, given that amelioration of these metabolic responses via the opening of cortical ATP-sensitive potassium (KATP) channels does not inhibit the nociceptor sensitization response (Zhao and Levy, 2018b). Whether COX-derived mediators released in the wake of CSD act directly on dural nociceptors to promote their mechanical sensitization or mediate this nociceptive effect by modulating meningeal immune cells remains to be examined. We also observed that local opening of K(ATP) channels ameliorates the prolonged activation, but not sensitization of dural nociceptors following CSD (Zhao and Levy, 2018b), further suggesting that the CSD-evoked activation and sensitization of dural nociceptors involve distinct mechanisms. The exact mechanism by which cortical K(ATP) channels affect the responses of dural nociceptors in the context of CSD remains to be elucidated.

## Cortical neurons, neuroinflammation, and meningeal nociception

Abnormal activation of cortical neurons and the subsequent development of parenchymal neuroinflammation were also proposed to drive meningeal nociception following CSD. Using a mouse model of CSD, Karatas et al. (Karatas et al., 2013) described a cascade of cortical events (Fig. 1) involving the opening of neuronal pannexin-1 channels, downstream caspase-1 activation, and HMGB1 release from the same neurons followed by nuclear factor-kB activation and increased parenchymal interleukin 1 beta (IL-1β) expression. This inflammatory cascade was further linked to the upregulation of COX-2 and inducible nitric oxide synthase in cortical astrocytes and the development of dural vasodilatation, suggesting a link between this cortical neuroinflammation and the activation of dural nociceptors. Other studies, however, failed to detect an acute release of HMGB1 (Takizawa et al., 2016) or upregulation of inducible nitric oxide synthase (Jander et al., 2001) following CSD, questioning the involvement of these inflammatory processes in mediating the meningeal vascular responses observed during the first hour after CSD. Takizawa et al. (2020) recently explored the development of cortical neuroinflammation in an optogenetic CSD model in mice, confirming upregulation of IL-1β at the level of their mRNA and also showing increases in interleukin 6 (IL-6) and C–C motif chemokine ligand 2 (CCL2) expression within the first-hour post CSD. The upregulation of IL-1β is of particular interest to the meningeal pronociceptive effect of CSD given its rapid increase as early as 10 min after CSD and its ability to promote both activation and mechanical sensitization of dural nociceptors (Zhang et al., 2012).

## CSD, cortical astrocytes, and meningeal nociception

CSD is associated with an acute activation of astrocyte signaling, including a robust wave of intracellular calcium (Ca^2+^) elevations (Chuquet et al., 2007, Enger et al., 2015, Peters et al., 2003, Zhao et al., 2021), which has been linked to the acute pial vascular response (Chuquet et al., 2007). Upon their activation, astrocytes release numerous proinflammatory agents, such as ATP, prostanoids, and cytokines/chemokines (e.g., IL-1β, CCL2) (Verkhratsky and Nedergaard, 2018) that could diffuse into the CSF-filled subarachnoid space, drive the pial vascular response and act upon leptomeningeal nociceptors (Fig. 1). The release of K^+^ from the glia limitans (Paulson and Newman, 1987) could also account, at least in part, for the acute activation of leptomeningeal afferents. We recently investigated the relative contribution of cortical astrocytes to the CSD-evoked dural nociceptor responses (Zhao et al., 2021). We found that inhibition of cortical astrocyte function, using two distinct pharmacological approaches, can suppress the CSD-evoked dural nociceptor sensitization but not the related activation of the nociceptors. Interestingly, we observed that the anti-nociceptive effect of the astrocyte inhibitors was not associated with inhibition of the CSD-related astrocyte Ca^2+^ wave, suggesting a mechanism involving Ca^2+^-independent astrocytic signaling. The notion that astrocytes can release arachidonic acid-derived prostanoids in a Ca^2+^-independent manner (Wang et al., 2020) and the finding that COX inhibition prevents the CSD-evoked sensitization of dural nociceptors points to a possible contribution of astrocyte-derived prostanoids as a critical underlying mechanism. Another astrocyte mediator that may contribute to the sensitization of dural nociceptors in the wake of CSD is ATP (Joseph et al., 2014). Astrocytes can release ATP through a Ca^2+^-independent process involving membrane pores, such as the P2X7 purinergic channel, pannexin-1, and anion channels (Kovács et al., 2018, Nikolic et al., 2020, Pan et al., 2015, Xiong et al., 2018). Extracellular ATP can augment ATP release from astrocytes via a mechanism involving connexin hemichannels (Davalos et al., 2005). ATP released by astrocytes may not directly act on dural nociceptors to promote their sensitization given that stimulation of meningeal purinergic receptors by exogenous ATP causes dural nociceptor activation (Zhao and Levy, 2015), a CSD-evoked nociceptive response not blocked by astrocytes inhibitors. However, it is possible that astrocyte-related ATP signaling contributes to the delayed sensitization of dural nociceptors indirectly by activating non-neuronal purinergic receptors such as P2X7 on microglia and meningeal immune cells, such as subdural macrophages (Di Virgilio et al., 2017, Van Hove et al., 2019).

## Cortex to meninges routes underlying meningeal nociception following CSD

The exact route mediators released in the cerebral cortex might take to reach the meninges following CSD remains unclear. Although the going theory is that cortical mediators released into the parenchymal interstitial space during the CSD phase transverse the pial layer via bulk diffusion and rapidly excite leptomeningeal nociceptors (Pietrobon and Moskowitz, 2013), such quick diffusion is limited by the glial limitans barrier that abuts the pial membrane. Under a steady-state condition, parenchymal interstitial fluid exits the brain via perivascular spaces; it enters the cerebrospinal fluid (CSF) that occupies the subarachnoid space utilizing the glymphatic transport mechanism (Hablitz and Nedergaard, 2021). This transport route could account for the activation of nerve endings localized near pial vessels and elsewhere within the subarachnoid space (Fricke et al., 1996, Liu-Chen et al., 1983, Mayberg et al., 1981, Uddman et al., 1981). However, the glymphatic transport route is unlikely to account for the acute activation of meningeal nociceptors because CSD is associated with an initial closure of perivascular spaces and reduced glymphatic flow (Schain et al., 2017). The gradual recovery of glymphatic flow during the 30 min post CSD, nonetheless, could explain a delayed activation of leptomeningeal afferents (Fig. 1). Algesic factors transported into the subarachnoid space or released in that space by subdural immune cells, however, are unlikely to influence dural nociceptors directly, given the physical separation of these meningeal spaces the arachnoid barrier layer and dural border cells (Coles et al., 2017). Another possible transport route of molecules in the CSF-filled subarachnoid space into the dural compartment involves zones near dural lymphatic vessels in peri-sinus dural regions (Fig. 1). These zones, potentially a part of the arachnoid granulation anatomical structure (Yagmurlu et al., 2021), have been suggested to serve as drainage entry points for CSF in the subarachnoid space into the dural lymphatic system (Louveau et al., 2018). Importantly, these peri-sinus dural areas are located near a dense population of chemosensitive dural nociceptors (Strassman et al., 2004). The relative slow transport of solutes from the subarachnoid space into the lymphatic drainage system (Louveau et al., 2018) could further explain the delay in the responses of dural nociceptors following CSD.

## Concluding remarks

The meningeal sensory system plays a key role in the genesis of the headache phase in migraine. Yet, the origin of its activation during a migraine attack is not well understood. In migraine with aura, the second-most common migraine subtype, the leading theory proposes that CSD is the pathophysiological event that underlies the aura phase and also leads to the activation of the meningeal sensory system. This theory is now strongly supported by preclinical studies of dural vascular changes as surrogate markers and direct electrophysiological recordings of dural nociceptors' responses.

Our current understanding of how CSD drives meningeal nociceptors remains incomplete, although recent data points to the roles of cortical neurons, astrocytes, and parenchymal neuroinflammation. However, there is very little support for the contribution of cortical vasodynamic and metabolic changes in driving meningeal nociception in the context of CSD. Preclinical data also suggest that the key components of neurogenic inflammation – vasodilatation and increased vascular permeability may not contribute to meningeal nociception following CSD. However, the recent finding of an inflammatory signal localized to the meninges overlying the occipital cortex in migraine patients experiencing visual aura and headache (Hadjikhani et al., 2020), and immune cell activation following CSD in mice (Schain et al., 2018) suggest an alternative meningeal inflammatory process in CSD-evoked migraine pain involving meningeal immunity.

While the most common type of migraine aura is visual, somatosensory auras are also reported (Viana et al., 2017), potentially resulting from CSD propagating into the somatosensory cortex (Bolay et al., 2019). The possibility that CSD can also occur in areas other than the visual or somatosensory cortices and give rise to a silent aura (Pietrobon and Moskowitz, 2013), thus serving as a general mechanism in migraine, was also proposed (Ayata, 2010). It should be emphasized, nevertheless, that despite the availability of direct and indirect evidence supporting a role for CSD as a noxious event that drives meningeal nociception, the views of CSD occurrence in migraine and its relevance to the headache phase are not universally accepted (Bolay et al., 2019, Charles, 2010). This notion is based primarily on the findings that some people with migraine may experience aura but not a headache, and evidence of therapeutic interventions that abolish aura but not headache (Wolthausen et al., 2009). Whether migraine involves CSD or other cortical hyperexcitability events, a better understanding of cortex to meninges signaling that might drive meningeal nociception could provide clues to the origin of the headache in migraine with and without aura. Increasing knowledge about the way CSD and perhaps other cortical events affect the responses of leptomeningeal afferents could have significant implications for the design of future pharmacological agents to treat migraine headache, in particular because their receptive fields in the subarachnoid space are not amenable to treatment with drugs that do not cross the blood-brain barrier, such as monoclonal antibodies.

## Declaration of Competing Interest

The authors declare that they have no known competing financial interests or personal relationships that could have appeared to influence the work reported in this paper.

## Data Availability

review, no data
